# Rates of False-Negative Screening in Prostate Specific Antigen Secondary to 5-Alpha Reductase Inhibitor Usage: A Quality-Improvement Initiative

**DOI:** 10.1590/S1677-5538.IBJU.2022.0099

**Published:** 2022-05-04

**Authors:** Justin Loloi, Matthew Wei, Mustufa Babar, Denzel Zhu, Ethan B. Fram, Pedro Maria

**Affiliations:** 1 Department of Urology Montefiore Medical Center Albert Einstein College of Medicine Bronx NY USA Department of Urology, Montefiore Medical Center, Albert Einstein College of Medicine, Bronx, NY, USA;; 2 Albert Einstein College of Medicine Bronx NY USA Albert Einstein College of Medicine, Bronx, NY, USA

**Keywords:** Prostatic Neoplasms, Prostate-Specific Antigen, 5-alpha Reductase Inhibitors, Prostatic Hyperplasia

## Abstract

**Purpose:**

Patients often take 5-alpha reductase inhibitors (5-ARIs) for the management of benign prostatic hyperplasia. However, 5-ARIs can decrease prostate specific antigen (PSA) by approximately half and therefore may lead to false negative PSA tests. We investigated false-screening rates in men on 5-ARIs undergoing PSA testing and whether ordering physicians noticed false negative findings.

**Materials and Methods:**

A single institution, retrospective study was conducted on patients with a PSA value documented between 2014 and 2017. Patient demographics, PSA results, 5-ARI usage, and providing clinician characteristics were collected. Published normal PSA values were used to determine PSA test positivity; values for those on 5-ARIs were doubled.

**Results:**

A total of 29,131 men were included. 1,654 (5.7%) were prescribed 5-ARIs at least 12 months prior to PSA evaluation. 118 men (7.1%) had a value that would be positive if corrected for 5-ARI usage, 33 (27.9%) of which had no indication that the provider had noted this. There was no effect on rates of false negative values if the PSA was ordered by a different provider than the one who prescribed the 5-ARI (p = 0.837). However, if the provider who ordered the PSA test was an urologist, the likelihood that a false negative value would be identified was lower (p=0.001).

**Conclusions:**

More than a quarter of men with false negative tests were missed. This occurred more often when the ordering provider was not an urologist. An educational opportunity exists to improve the quality of PSA testing by preventing false negative tests.

## INTRODUCTION

Prostate cancer (PCa) is the most commonly diagnosed and second most lethal cancer of men in the United States ( 1 , 2 ). Despite the recent controversy and discovery of additional novel biomarkers for PCa, prostate specific antigen (PSA) remains the most widely used tool for PCa screening and plays a key role in decreasing mortality from the disease ( 1 , 3 ). Patients with PCa often also present with the comorbidity of benign prostatic hyperplasia (BPH), an exceedingly common condition affecting the aging male population ( 4 ). BPH is frequently managed with 5-alpha reductase inhibitors (5-ARI): finasteride and dutasteride. 5-ARIs inhibit the production of dihydrotestosterone and reduce prostate gland size and vascularity, thereby improving lower urinary tract symptoms (LUTS) ( 5 - 7 ).

Although 5-ARIs are effective in treating BPH, there are rising concerns regarding its usage in patients being screened for PCa. 5-ARIs not only decrease DHT but also systemic levels of PSA by about half ( 8 ) which may delay detection and intervention in cases of undiagnosed PCa. Doubling PSA values has been a technique used to account for decreased levels due to 5-ARI treatment and has been shown to increase the sensitivity of PSA for PCa diagnosis ( 9 ). However, certain clinicians may not routinely implement this technique in clinical practice, as they may be unaware of 5-ARIs’ suppressive effects on PSA ( 8 , 10 ). This study sought to determine the false-screening rate in men on 5-ARIs undergoing PSA testing and determine whether ordering physicians had noticed these false negative findings. We hypothesized a high false-screening rate in men on 5-ARIs undergoing PSA testing and that these rates would be higher if the PSA was ordered by a non-urologist when compared to a urologist.

## MATERIALS AND METHODS

### Study Design and Patient Population

After obtaining IRB approval (IRB#2013-2712), we conducted a cross-sectional study of all patients who had PSA values at our academic hospital institution (which provides comprehensive primary care and urologic care) from January 2014 to July 2017. Using Clinical Looking Glass (Streamline Health, Atlanta, GA), a system of querying our institutional database of electronic medical records, we built a cohort of adult patients who had a PSA test ( 11 ) and excluded those with any history of PCa. Then, we examined the cohort for prescriptions for 5-ARIs within 12 months prior to the PSA test, and also collected patient demographics (e.g. age at PSA test, self-reported race/ethnicity, preferred language), clinical characteristics (e.g. PSA value, 5-ARI type (finasteride vs. dutasteride)), and whether the physician who ordered the PSA test was an urologist or non-urologist.

Among the subset of patients with a 5-ARI prescription, we determined if the physician who ordered the PSA test was the same physician who prescribed the 5-ARI. When determining PSA test positivity, we utilized published normal values per age, in which the cutoff values for a positive PSA for men aged <50, 50-59, 60-69, and 70-79 was 2.5, 3.5, 4.5, and 6.5 ng/mL, respectively ( 12 ). Parameters including PSA density and percentage of free PSA were not used to determine PSA positivity. For men with a 5-ARI prescription, PSA results were doubled ( 13 ). A PSA test was considered to be a false negative if no subsequent workup (ex. repeat PSA, prostate biopsy) was ordered when the adjusted PSA result was positive. Manual chart review was conducted to determine if the physician who ordered the PSA test was aware of the effect of the 5-ARI.

### Statistical Analysis

Categorical variables were compared using the χ2-test and continuous, normally distributed and non-normally distributed variables were compared using the independent samples t-test and the Mann-Whitney *U* test, respectively. We then constructed 2x2 tables comparing false negative rates among patients taking a 5-ARI, based on whether an urologist had ordered the 5-ARI, and whether the physician who prescribed the 5-ARI had ordered the PSA test. All statistical tests were two-sided with a significance threshold of p≤0.05. All analysis was conducted in Stata v16.1 (StataCorp, College Station, TX).

## RESULTS

**Figure 1:**
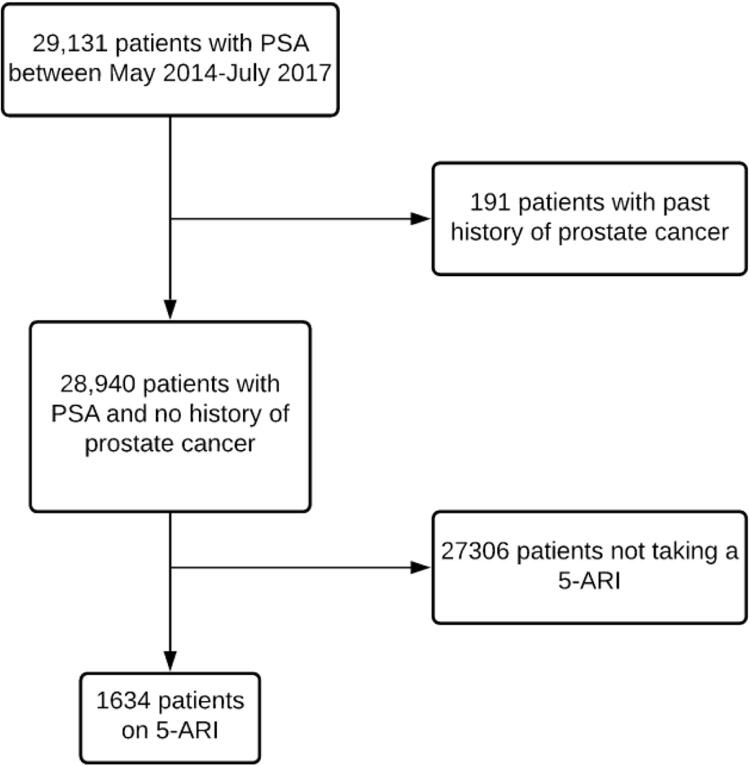
Flow chart of participants in cohort study.

A total of 29,131 men met inclusion criteria, 191 of which were excluded due to a history of PCa. Therefore, the total cohort consisted of 28,940 men ( Figure-1 ). Of the 28,940 men, 1,654 (5.7%) were reported as being prescribed a 5-ARI in the 12 months prior to the incident PSA screening test ( Table-1 ). Men who took 5-ARIs were typically older (mean age 69.5±10.5 yrs) compared to men who did not take 5-ARIs (58.9±10.8 yrs, p<0.00001). Additionally, the proportion of non-Hispanic White (NHW) men were higher among those on 5-ARIs (22.2%) when compared to NHW men not on 5-ARIs (13.9%, p<0.0001).

**Table 1:** Patient and clinical characteristics of all patients, stratified by 5-alpha reductase inhibitor (5-ARI) use.

	All patients	5-ARI		

Characteristics		No	Yes	
			
	N=28,940	N=27285 (94.4%)	N=1634 (5.7%)	p
Age at PSA Test, mean, SD (yrs)	59.5, 11.1	58.9, 10.8	69.5, 10.5	<0.00001
**Age category, N (%)**				<0.0001
18-39.9	561 (2.0)	548 (2.0)	12 (0.7)	
40-49.9	4574 (15.8)	4526 (16.6)	46 (2.8)	
50-59.9	9912 (34.3)	9710 (35.6)	197 (12.1)	
60-69.9	8646 (29.9)	8098 (29.7)	541 (33.1)	
≥70	5247 (18.1)	4403 (16.1)	838 (51.3)	
**Self-reported Race/Ethnicity, N (%)**				<0.0001
Non-Hispanic White	4161 (14.4)	3798 (13.9)	363 (22.2)	
Non-Hispanic Black	8870 (30.7)	8417 (30.9)	453 (27.7)	
Hispanic	9062 (31.3)	8573 (31.4)	489 (29.9)	
Others/Declined*	6826 (23.6)	6497 (23.8)	329 (20.1)	
**Preferred Language, N (%)**				<0.0001
English	23143 (80.0)	21894 (80.2)	1249 (76.4)	
Spanish	4718 (16.3)	4394 (16.1)	324 (19.8)	
Others/Declined	1079 (3.7)	997 (3.7)	61 (3.7)	
PSA, median (IQR) (ng/mL)	0.94 (0.50-2.0)	0.9 (0.5-1.9)	2.2 (0.9-5.4)	
**Urologist ordered PSA, N (%)**				<0.0001
No	26285 (90.8)	25035 (91.8)	1230 (75.3)	
Yes	2655 (9.2)	2250 (8.3)	404 (24.7)	

P value refers to independent samples T-test (age) or χ^2^-test (categorical variables).

*Includes Asians and American Indians/Alaskan Natives, which made up <3% of the total population.

**Includes Dutasteride in combination with Tamsulosin.

Among the 1,654 men on 5-ARIs, 118 (7.1%) had a PSA value that would be positive if corrected for 5-ARI use ( Table-1 ). Furthermore, among the 1,654 men, those with a false negative PSA were more likely to be prescribed dutasteride as their 5-ARI (21, 18.1%) when compared to those without a false negative PSA (272, 17.9%, p=0.0025). There was no significant difference in age at PSA test, race/ethnicity, and preferred language of men with a false negative PSA when compared to those without a false negative PSA.

Of the 118 men with a false negative PSA value, 33 (27.9%) had no indication that the provider had noted the false negative result ( Table-2 ). However, there was an increase in the likelihood that a false negative value would be identified if the provider who ordered the PSA test was an urologist than if the provider was a non-urologist (p=0.001). There was no significant difference in the identification of false negative rates if the PSA test was ordered by a different provider than the one who prescribed the 5-ARI (p=0.837).

**Table 2:** A) Patient and clinical characteristics of patients on 5-ARI, stratified by whether they had a false negative value, or not, and B) observed false negative rate among patients treated with 5-ARI, if the PSA was ordered by an urologist vs non-urologist or (C) if the PSA was ordered by the same clinician who ordered the 5-ARI.

	All patients	False Negative		

A. Characteristics		No	Yes	
			
	N=1634	N=1518 (92.9%)	N=118 (7.1%)	p
Age at PSA Test, mean, SD (yrs)	69.5, 10.5	69.6, 10.7	68.6, 7.1	0.31
**Age category, N (%)**				0.13
18-39.9	12 (0.7)	12 (0.7)	0 (0)	
40-49.9	46 (2.8)	43 (2.8)	3 (2.6)	
50-59.9	197 (12.1)	189 (12.5)	8 (6.9)	
60-69.9	541 (33.1)	492 (32.4)	49 (42.2)	
≥70	838 (51.3)	782 (51.5)	56 (48.3)	
**Self-reported Race/Ethnicity, N (%)**				0.96
Non-Hispanic White	363 (22.2)	339 (22.3)	24 (20.7)	
Non-Hispanic Black	453 (27.7)	420 (27.7)	33 (28.5)	
Hispanic	489 (29.9)	455 (30.0)	34 (29.3)	
Others/Declined*	329 (20.1)	304 (20.0)	25 (21.6)	
**Preferred Language, N (%)**				0.21
English	1249 (76.4)	1166 (76.8)	83 (71.6)	
Spanish	324 (19.8)	294 (19.4)	30 (25.9)	
Others/Declined	61 (3.7)	58 (3.8)	3 (2.6)	
PSA, median (IQR) (ng/mL)	0.94 (0.5-2.0)	0.94 (0.5-1.9)	3.5 (2.9-4.5)	<0.00001
**5-ARI Type**				0.0025
Dutasteride**	293 (17.9)	272 (17.9)	21 (18.1)	
Finasteride	1341 (82.1)	1246 (82.1)	95 (81.9)	
	N=118	N=33 (27.9)	N=85 (72.1)	
		
**B. Urologist**				0.001
No	81 (68.6)	30 (90.9)	51 (60.0)	
Yes	37 (31.4)	3 (9.1)	34 (40.0)	
**C. Concordant**				0.837
No	67 (56.8)	18 (54.5)	49 (57.6)	
Yes	51 (43.2)	15 (45.5)	36 (42.4)	

P value refers to independent samples T-test (age) or χ^2^-test (categorical variables).

*Includes Asians and American Indians/Alaskan Natives, which made up <3% of the total population.

**Includes Dutasteride in combination with Tamsulosin

## DISCUSSION

To our knowledge, this study is the first to analyze the rates of false negative PSA tests during 5-ARI therapy in patients under two scenarios: PSA tests ordered either by an urologist vs non-urologist, and concordance in providers prescribing 5-ARI and ordering PSA screenings. Our study found that there are significantly more missed false negative tests when the ordering provider is a non-urologist but no difference when looking at concordance of care.

5-ARIs represent a first line medical therapy for patients with benign prostatic enlargement. Multiple studies have supported their safety and efficacy in treating BPH related symptoms and increasing PSA test sensitivity for PCa if interpreted correctly ( 14 - 16 ). The doubling of PSA values for PCa screening has been an effective technique used to correct for decreased levels in patients taking 5-ARIs, although alternative strategies have been suggested, such as a PSA increase from nadir >0.3 ng/mL ( 17 ). However, non-urologists may not be aware of this practical rule, especially since the American Society of Clinical Oncology, American Urological Association, and National Comprehensive Cancer Network Prostate Cancer Early Detection do not clearly state a PSA cutoff in men taking 5-ARIs to indicate prostate biopsy ( 18 , 19 ). Consequently, increasing providers’ awareness of doubling PSA may increase its effectiveness as a viable tool for men undergoing PCa screening.

There have been concerns regarding 5-ARI use and PCa outcomes. Multiple studies have found that the use of 5-ARIs is associated with delayed diagnosis and increase in PCa mortality ( 10 , 20 ). Recently, Busato et al. ( 8 ) expressed their concerns that in Brazil, 5-ARIs are often prescribed by non-urologists and that about 90% of PSA screening tests are ordered by primary care physicians while only 7% are ordered by urologists. In our study, of the total patients who were taking 5-ARIs, 75% were prescribed by non-urologists and 75% of PSA screening tests were ordered by primary care physicians. Therefore, our study also supports that physician prescribing the 5-ARIs and ordering PSA tests are often non-urologist who may not be aware about 5-ARI induced PSA suppression. It should be noted that a positive PSA should be confirmed after a few weeks under standardized conditions, such as ejaculation, manipulations, or urinary tract infections, in the same laboratory before considering further interventions ( 21 ). Although multiple interventions to improve the issue at hand can be considered, our study justifies a concerted effort in educating non-urologists who prescribe 5-ARIs and order PSA tests.

A systematic review of adverse effects and safety of 5-ARIs conducted by Hirshburg et al. ( 22 ) in 2016 summarized that although there is no increase in incidence of PCa, there is an increased risk of high-grade PCa when detected. They did not find negative impact on the survival rates of patients with PCa who had a history of 5-ARI use. While it is possible that 5-ARI use could make patients more susceptible to develop high-grade disease, it is also plausible that 5-ARI use delays PCa detection, with patients subsequently presenting with higher stage disease due to seemingly normal screening; however, further studies should investigate these specifics and the possibility of both contributing factors should be considered.

The results from our study create an opportunity for intervention through education and integration of computerized clinical decision support tools. Professional organizations, including the American Society of Clinical Oncology, American Urological Association, and National Comprehensive Cancer Network Prostate Cancer Early Detection, can join efforts in creating specific guidelines in interpreting PSA values in men taking 5-ARIs. Additionally, clinical decision support technology tools integrated into electronic health record softwares have demonstrated to reduce medical errors and improve patient outcomes across a variety of health care settings ( 23 , 24 ). Therefore, a potential intervention is the integration of corrected PSA values in men using 5-ARIs into electronic health record softwares in order to improve accuracy of PCa risk assessment and biopsy referral.

This study is not without limitations. The retrospective nature and involvement of a single center can introduce selection bias and decrease generalizability. Additionally, there were small sample sizes in some of the cohorts and thus there could be shifts in statistical significance with larger sample sizes. Furthermore, while 5-ARIs are well documented to decrease PSA levels, there are also other medications that we did not control for, including non-steroid anti-inflammatory drugs, statins, and thiazide diuretics that have also been shown to decrease PSA levels up to 36% ( 25 ). Additionally, there are other factors that can affect PSA, such as prostate volume, BPH, and prostatitis, that were not controlled for in the study. Nonetheless, despite these limitations, we believe our data offers insight into the importance of considering whether patients are on 5-ARIs during PSA screening. This group is working on a subsequent study aiming to delineate real-time physician practice in the community, focusing on the patterns and trends in PSA screening and 5-ARI prescribing.

## CONCLUSION

Despite their important role in the treatment of BPH, 5-ARIs may contribute to false-negative PSA screening tests. Non-urologists had missed more false negative tests compared to urologists, however, there was no difference in noticed rates of false negative tests when we examined concordance of care. Given the considerable morbidity and mortality associated with PCa, we recommend community-wide efforts to further educate clinicians on the effects of 5-ARIs on PSA levels.
